# What Are the Key Factors of Functional Outcomes in Patients with Spinopelvic Dissociation Treated with Triangular Osteosynthesis?

**DOI:** 10.3390/jcm11226715

**Published:** 2022-11-13

**Authors:** Po-Han Su, Yi-Hsun Huang, Chen-Wei Yeh, Chun-Yen Chen, Yuan-Shun Lo, Hsien-Te Chen, Chun-Hao Tsai

**Affiliations:** 1 School of Medicine, China Medical University, Taichung 404, Taiwan; 2 Department of Orthopedic Surgery, China Medical University Hospital, China Medical University, Taichung 404, Taiwan; 3 Department of Orthopedic Surgery, Wei Gong Memorial Hospital, Miaoli 351, Taiwan; 4 Department of Orthopedic Surgery, China Medical University Bei Gang Hospital, Yunlin 651, Taiwan; 5 Department of Sports Medicine, College of Health Care, China Medical University, Taichung 404, Taiwan

**Keywords:** spinopelvic dissociation, triangular osteosynthesis, functional outcome

## Abstract

For patients with spinopelvic dissociation (SPD), triangular osteosynthesis is the current method for the fixation of the posterior pelvis. This study aimed to assess the recovery process and radiographic parameters associated with the functional outcomes in patients with SPD treated by triangular osteosynthesis. We collected data from 23 patients with SPD. To investigate the key aspect regarding the functional outcomes of these patients, we measured pre- and post-operative parameters, and a statistical analysis adjusted for age, gender, and time windows was used. The radiographic displacement measurement in the pre-operative period showed that the EQ−5D−5L increased by 2.141 per outlet ratio unit. The EQ−5D−5L increased by 1.359 per inlet ratio unit and 1.804 per outlet ratio during the postoperative period. The EQ−VAS increased significantly only with the inlet ratio in the postoperative period (1.270 per inlet ratio). A vertical reduction in SPD during the surgery can achieve more satisfactory outcomes than a horizontal anatomical reduction, in which the horizontal displacement causes inferior functional outcomes.

## 1. Introduction

Spinopelvic dissociation (SPD) is associated with transverse sacral fractures, which cause the dissociation of the sacrum from the pelvis [1,2]. It is associated with 3% of transverse sacral fractures and 3% of sacral fractures are associated with pelvic ring injuries [3]. SPD is well known for its high mortality and comorbidities such as nerve root injuries [4]. When SPD is correctly diagnosed and appropriately treated, patient outcomes can be optimized [5]. However, a high level of consensus and a unified approach for dealing with this complex issue are lacking.

**Figure 1:**
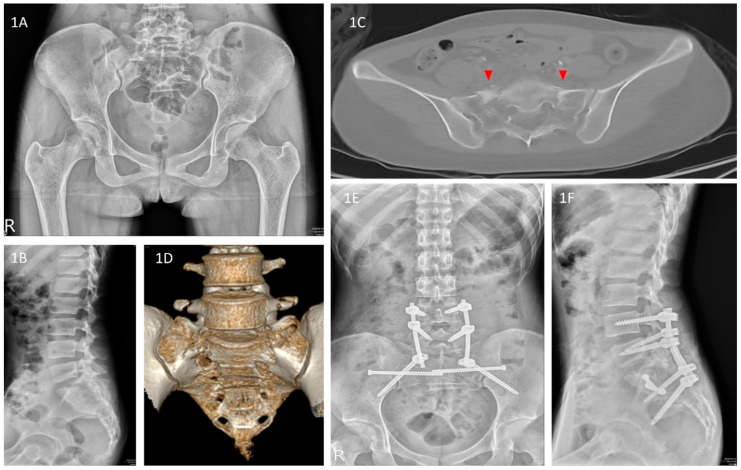
(**A**–**F**) Pre- and postoperative radiography and CT images of a 21-year-old woman who fell from a bridge and developed bifrontal EDH, facial bone fractures, and bilateral sacral fractures with spinopelvic dissociation that were presented in the emergency room. (**A**) AP view of the pelvis on admission showing bilateral fracture lines on the sacrum; (**B**) lateral view of the sacral spine view on admission showing fracture lines on the sacrum, indicating displaced fragments over the fracture site; (**C**) axial CT view of the sacrum demonstrating bilateral fracture lines indicating U-type sacral fractures; (**D**) 3D CT view of the sacrum demonstrating a displaced U-shape sacral fracture (red arrows) and AO/OTA 54C3 type, Denis Zone II sacral fracture; (**E**) Postoperative AP pelvic view: the sacral fracture was stabilized by bilateral triangular osteosynthesis with S2AI screws. (**F**) Postoperative lateral view of the sacral spine: The trajectory of S2AI screws was set under O-arm navigation to obtain an optimal length. Abbreviations: EDH = epidural hematoma, CT = computed tomography, and AP = anteroposterior.

The traditional fixation methods for the posterior pelvic ring include tension band transiliac plate fixation, local plate fixation, open or percutaneous ilio-sacral screw fixation, and transiliac bars, which do not guarantee postoperative stability and may result in fixation failures [6,7]. In recent years, surgeons have used triangular osteosynthesis (TOS) in combination with the surgical technique of unilateral L5 fixation using S2AI or iliac screws for SPD treatment, and the literature indicates that these patients show satisfactory postoperative function and radiological outcomes [8]. With or without a combination of bilateral or dual iliac screw fixation techniques [9], TOS is a reliable form of fixation that enables early weight-bearing while preventing the loss of reduction [9,10,11,12]. In addition, compared with traditional surgical methods, its complication rate is low [3,5] (Figure 1A–F).

Currently, a radiographic assessment remains the standard peri-operative measurement for displacement and reduction in studies of pelvic fractures. However, there is still a lack of research investigating the relationship between peri-operative SPD and prognosis from the perspective of radiology in patients with SPD who underwent reduction and fixation by TOS using S2AI screws. Though the measurement of outcomes is difficult and the level of evidence in this area is poor, this article revealed three such methods for measuring radiographic displacement [11,13,14].

This study aimed to investigate the recovery time course and imaging parameters relevant to the functional recovery of patients with SPD treated by TOS.

## 2. Materials and Methods

### 2.1. Patient Selection and Classification

**Figure 2:**
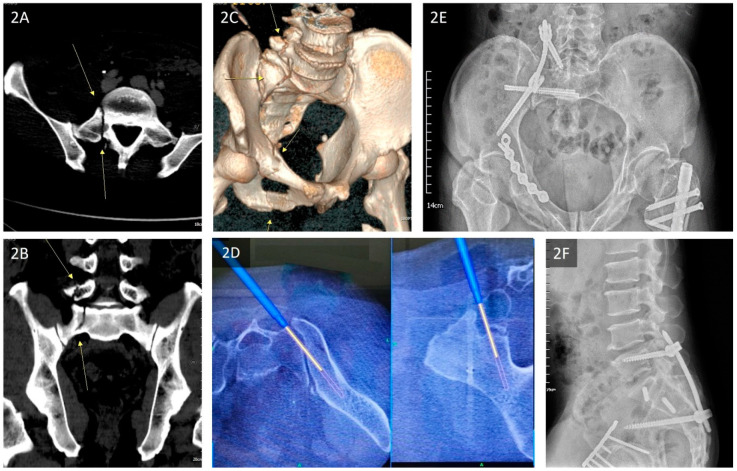
(**A**–**F**) Pre- and postoperative radiography and CT images of a 56-year-old man who was hit by a vehicle and had right superior and inferior rami fractures, a right L5 transverse process fracture, and a right sacral fracture with spinopelvic dissociation. The associated injury included a left femoral shaft fracture, right femoral shaft segmental fracture, and left medial malleolar fracture. (**A**) Axial CT view of the sacrum on admission demonstrating fracture lines (yellow arrows) over the right L5 transverse process; (**B**) coronal CT view of the sacrum on admission demonstrating fracture lines (yellow arrows) over the right sacrum; (**C**) 3D CT view of the pelvis showing right superior and inferior rami fractures (yellow arrows), a right L5 transverse process fracture (yellow arrows), and a right AO/OTA 61C1.3 and 54B3 type, Denis Zone II, sacral fracture (yellow arrows); (**D**) with the assistance of O-arm navigation, the optimal trajectory of the S2AI screw was set (O-arm and Stealth Station S7 Surgical Navigation System, Stryker); (**E**) postoperative view of the inlet pelvis—the sacral fracture was stabilized by triangular osteosynthesis with S2AI screws, and the right superior rami fracture was reduced and fixed with a pre-contoured locking plate; (**F**) postoperative lateral view of the sacral spine. Abbreviations: CT = computed tomography.

This is an observational, retrospective study. From August 2018 to September 2021, 29 patients with SPD were recruited. One was excluded due to severe spinal cord injuries, and five were lost to follow-up in our orthopedic clinic department. Complete series of pre-and post-operative radiographs were collected from the remaining 23 patients who suffered pelvic fractures with SPD treated by TOS fixation using the S2AI screw fixation technique. To make the procedure more appropriate and to obtain an optimal length and deflection angle, we set the trajectory of the S2AI screws under O-arm navigation (Figure 2A–F) [15]. These patients were postoperatively followed-up for a minimum of one year in the clinic as a single cohort. The study protocol was approved by the Research Ethics Committee of the China Medical University Hospital, Taichung, Taiwan (protocol ID: CMUH108−REC3−144) and conducted in accordance with the ethical principles of the Helsinki Declaration. The inclusion criteria include skeletally mature patients who suffered pelvic fractures with SPD treated by TOS fixation. Based on the anatomic relationship between the fracture site and the sacral neural foramen, Denis et al. classified sacral fractures into three types. Roy-Camille et al. classified transverse sacral fractures of the Denis III zone into three subtypes based on the degree of displacement and the traumatic mechanism [16,17]. In this study, most patients were in Denis zones I and II.

### 2.2. Radiographic Methods

To assess the imaging parameters associated with the functional recovery, we used three radiographic methods and three functional outcome questionnaires in this study.

Three experienced orthopedic trauma surgeons collaborated on this measurement plan and independently measured radiographic features. For measurements using computer-based-reading methods, each observer was given an identical set of images (pre- and postoperative anteroposterior (AP), outlet, and inlet views). This study aimed to investigate the correlation between radiological measurements and the functional outcome. Three previously published radiographic measurement methods were chosen. Each observer was provided with a set of images (23 patients and six images per patient) and received the same instructions for measurement, including three radiographic measurement methods, which are described below (Table 1).

**Table 1:** Radiographic measurement methods for assessment of displacement and symmetry for pelvic fracture with spinopelvic dissociation.

Authors	Methods	Description
Sagi et al., 2009	Inlet and outlet ratio Method (Sagi Method) [11]	On the inlet view, we drew a line across the anterior border of the sacrum, perpendicular to the spinous processes. The perpendicular distance from this line to the subchondral bone of each acetabulum was measured, and a ratio was then calculated, with the affected side of pelvis set as the numerator. A similar ratio was obtained for the outlet view by drawing a line parallel to the superior end plate of S1, perpendicular to the spinous processes. The perpendicular distance from the reference line to the subchondral bone of each acetabulum was measured, and a ratio was then calculated, with the affected side of pelvis set as the numerator (Figure 3).
Keshishyan et al., 1995	Cross measurement method (Keshishyan Method) [14]	The measurement method described by Keshishyan et al. for assessing the displacement of pelvic ring continuity in children used only the AP pelvic view. Originally, this method was applied for skeletally immature patients and measures the distance from the inferior aspect of the sacroiliac (SI) joint to the contralateral triradiate cartilage. We used the modified method described by Lefaivre et al. to assess our adult patients. Observers were instructed to measure from the inferior SI joint (iliac side) to the inferior aspect of the teardrop in the AP pelvic view. “Y” was the length from the left SI joint to the right teardrop, and “X” was the opposite. Observers were instructed to measure the distance using the measuring software. We then calculated the ratio (X/Y) to standardize the baseline of comparison of the displacement (Figure 4).
Lefaivre et al., 2009	Absolute displacement method (ADM) [13]	This method was initially proposed by Lefaivre et al. in 2009. Observers were instructed to use preoperative pelvic AP, inlet, and outlet views. In each view, a horizontal line was drawn across the superior end plate of L5 as a reference line. If this was not visible in the film, the observers were asked to use the inferior end plate of L5 as a reference. Measurements were either parallel or perpendicular to this reference line. This line was used as the direction for horizontal measurements, or a line 90 degrees to this reference line was used for vertical measurements. Maximum displacements in the anterior and posterior pelvic rings were measured in each plane film. After completing the six measurements of the three preoperative films (anterior and posterior rings in each of the AP, outlet, and inlet views), the observers were instructed to measure the same anatomic locations in the postoperative plane films. Finally, the largest single measurements from the six preoperative and postoperative measurements were considered the preoperative and postoperative maximum displacements, respectively (Figure 5A,B).

**Figure 3:**
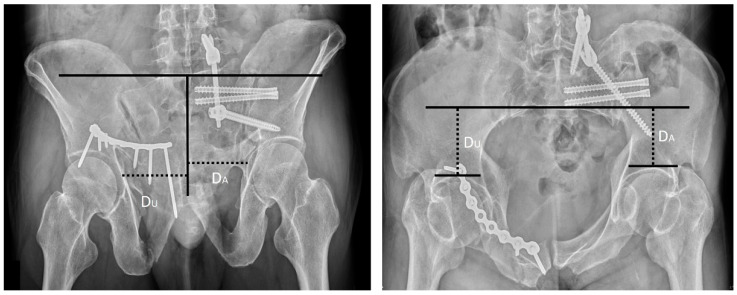
The inlet and outlet ratio are calculated (DA/DU) with the pelvic inlet and outlet views. The solid lines refer the reference lines, and the dashed lines refer measured lines. The Abbreviations: DA = distance of affected side; DU = distance of unaffected side.

**Figure 4:**
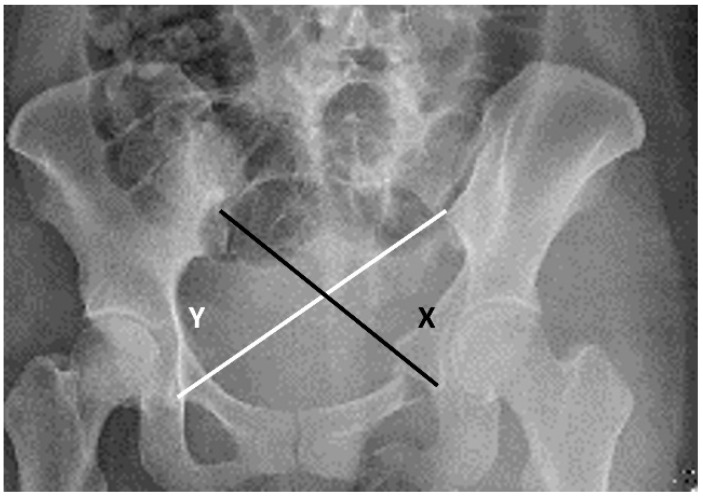
The cross−measurement method is illustrated with an example image.

**Figure 5:**
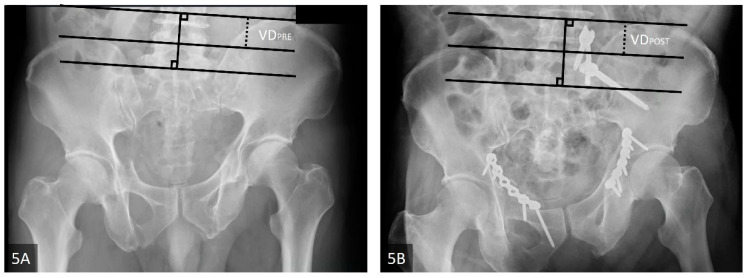
(**A**,**B**) The absolute displacement method (ADM). The example image illustrated the measurement of vertical displacement in pelvis AP view pre- and post-operatively. (**A**) Pre-operative pelvis AP view. (**B**) Post-operative pelvis AP view. The reference lines are solid, and the measured lines are dashed. The Abbreviations: VDPRE = the pre-operative vertical displacement; VDPOST = the post-operative vertical displacement.

### 2.3. Statistical Methods

Numbers (percentages) were used to represent the distribution of gender, AO 2018 classification, and Denis zone [16]. The mean (standard deviation [SD]) and median (interquartile range [IQR]) were used to show the distribution of age, the radiographic displacement measurement (including the inlet ratio, outlet ratio, deformity index, asymmetry, deformity ratio, vertical displacement [VD], and horizontal displacement [HD]) in pre- and postoperative periods, VD change (preoperative minus postoperative values), and HD change (preoperative minus postoperative values). Generalized estimating equations (GEE) were used to estimate differences in outcomes (including the EQ−5D−5L [18], EQ−VAS, and Majeed pelvic scores [19]) among different time windows. The model was adjusted for age and gender. We also used the GEE model to assess the association between outcomes and different radiographic displacement measurements. The model was adjusted for age, gender, and time windows.

## 3. Results

As shown in Table 2, a total of 23 patients were enrolled in this study. There were 15 men and 8 women (65.2% vs. 34.8%), and the mean age was 47.8 (19.3) years. More than half of the patients were in the 61C1 (60.9%) category according to the AO 2018 classification, followed by those in the 61C3 (26.1%), 61C2 (8.70%), and 62C2 (4.35%) categories.

**Figure 6:**
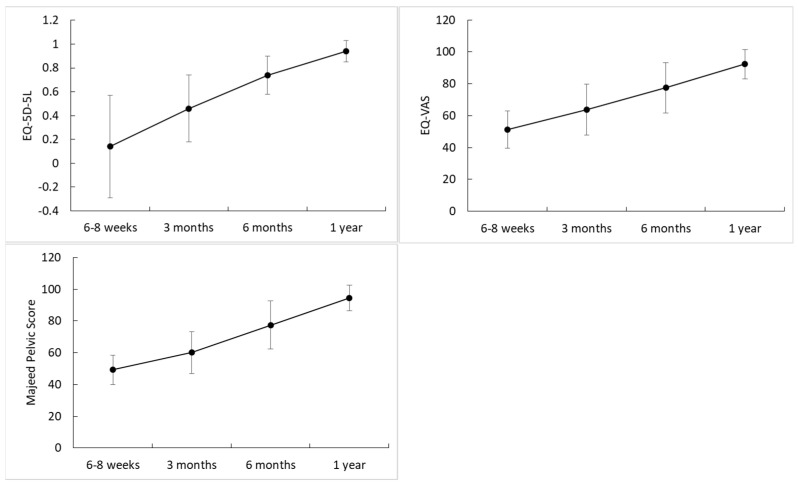
EQ−5D−5L, EQ−VAS, and Majeed pelvic scores over time.

As time progressed, the functional outcomes improved, and the patients returned to a near-normal life within one year. The EQ−5D−5L score increased with time, from 0.14 at 6–8 weeks to 0.94 at one year. The differences for the time trend were 0.32 in the crude GEE model (95% confidence interval [CI]: 0.25, 0.39) and 0.31 in the adjusted GEE model (95% CI: 0.25, 0.37) (Table 3, Figure 6). The EQ−VAS and Majeed pelvic scores also increased with time. The differences for the time trend were 0.17 for the EQ−VAS (95% CI: 0.14, 0.30) and 0.20 for the Majeed pelvic score in the adjusted GEE model (95% CI: 0.18, 0.22).

In this study, three image-evaluation methods, including the measurement of the inlet–outlet ratio, the cross−measurement method, and ADM, were used pre-and postoperatively. The association between the EQ−5D−5L score and the radiographic displacement measurement is presented in Table 4. For the preoperative radiographic displacement measurements, the EQ−5D−5L score increased by 2.141 per outlet ratio unit (95% CI: 0.041, 4.241). In the postoperative period, the EQ−5D−5L score increased by 1.359 per inlet ratio unit and 1.804 per outlet ratio (95% CI: 1.301, 2.307) but decreased by 0.01 per HD (95% CI: −0.018, −0.002) after adjusting for age, gender, and the follow-up time. This shows that changes in the horizontal direction are more correlated with EQ−5D−5L recovery.

The association between the EQ−VAS score and the radiographic displacement measurements is shown in Table 5. The association was significant only with the inlet ratio in the postoperative period. The EQ−VAS score increased by 1.270 per inlet ratio (95% CI: 0.093, 2.447) in the adjusted GEE model. However, there were no significant associations between the Majeed pelvic score and any of the radiographic displacement measurements (Table 6).

## 4. Discussion

The present study revealed that the displacement of SPD in spinopelvic fixation provides good vertical reduction results. During surgery, a reduction in the vertical direction is easier to achieve by fluoroscopy. A vertical anatomical reduction is often mentioned and highlighted for the treatment of unequal feet. A vertical displacement causes differences in the lower extremities, abnormal motor gaits, and lower Majeed scores.

It is sometimes difficult to achieve a perfect horizontal reduction due to comminuted sacroiliac fractures or an indirect reduction in the sacroiliac joints with complex anatomical structures radiologically.

**Figure 7:**
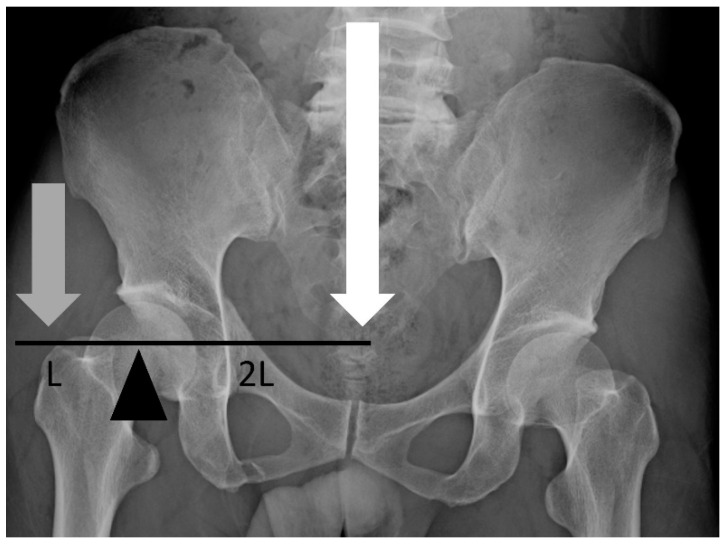
Coronal view of the hip illustrating the change of the lever arm around the hip’s center. When we set the hip’s center as a fulcrum, there are two opposing forces across the hip’s center: the body weight (white arrow) and abductor muscles (gray arrow). The lever arm for body weight is longer. In patients with SPD, horizontal displacement of the hip’s center often extends the lever arm for body weight, causing increased work to balance the moment.

Regarding horizontal reduction, the analysis showed that patients with a short-term follow-up showed a lower tolerance for postoperative horizontal displacement. Only a few studies have focused on the relationship between the inferior quality of horizontal displacement reduction and unsatisfying functional outcomes. We believe that the inferior quality of the horizontal reduction results in a change in the lever arm of the peak moment of the hip, which causes greater work in terms of hip abduction, adduction, flexion, and extension in the affected side in patients with SPD (Figure 7). As a result, the centroid experiences a mid-lateral shift, which may increase the metabolic cost and mechanical work of the lower extremities [20,21]. With rehabilitation, patients improved their function over time, but the change of the lever arm may contribute to unsatisfaction, increasing metabolic costs, and increased mechanical work in the short term postoperatively. No significant correlation was found in the asymmetric index. This could be because the integrity of the pelvic ring was restored postoperatively, while the SI joint was left without a complete reduction.

Our results revealed that the patient’s function will return to normal in one year. The postoperative gait analyses of patients with various pelvic ring fractures by Kubota et al. [22,23] showed that there was a complete recovery of peak hip abduction, and a partial recovery of peak hip extension and hip strength were noted at the 12-month follow-up. The horizontal displacement of the pelvis may affect the offset change of the hip joint, which is associated with abductor function. Dean et al. [24] concluded that patients with type C pelvic fractures had weaker hip abductor strengths, lower peak hip abduction moments, slower walking speeds, lower peak hip abductions, and lower peak hip extensions in the short-term after the surgery; however, at the 12-month follow-up, the bilateral hip strength (abduction, adduction, flexion, and extension), bilateral peak hip moment (abduction, adduction, flexion, and extension), peak hip power, or walking speed did not differ between groups. We reasoned that an insufficient hip abduction strength may in turn lead to differences in short-term functional outcomes [22,23,24].

There is no perfect assessment tool, and the measurements of pelvic radiographs have not yet been well validated [25]. This is the first study to connect functional outcomes to radiological assessments. We found that the questionnaires and assessment tools for functional outcomes were often subjective and generalized; therefore, currently, we can hardly ascribe the unsatisfying hip function to the postoperative horizontal residual displacement. Although reduction is important, the evaluation of the association between the radiological displacement and functional outcomes requires better tools. In patients with SPD, there will be multi-axial displacements, including horizontal, vertical, and rotational displacements. The plain radiographs could only reveal the measurement of horizontal or vertical displacements, whereas the rotational displacement could be assessed by CT. It is reported that CT or three-dimensional reconstruction-based displacement measurements of pelvic ring injury displacement may provide a more accurate assessment [26].

This study had some limitations. First, a tomographic analysis is warranted to assess the rotation, but a customized view along the long axis of the pelvic bone is required for a correct assessment. Second, this was a single-center, observational, retrospective study with a small number of participants. However, as patients suffering from pelvic fractures with SPD are relatively rare, greater-scale research is difficult to carry out. To improve patients’ functional outcomes and satisfaction, this study sets a template for future research focusing on this topic. Further studies with more patient data would help to improve the understanding of the correlation between the functional outcomes and reductions in different dimensions.

## 5. Conclusions

TOS is a powerful fixation technique for patients with SPD. We achieved the vertical reduction in SPD more easily through fluoroscopy during the operation than with horizontal anatomical reduction, while horizontal displacement caused inferior satisfaction.

## Figures and Tables

**Table 2:** Patients’ characteristics (*n* = 12).

Variable	*n* (%)	
Men	15 (65.2%)	
Women	8 (34.8%)	
AO 2018 classification		
61C1	14 (60.9%)	
61C2	2 (8.70%)	
61C3	6 (26.1%)	
62C2	1 (4.35%)	
Denis zone		
I	10 (43.5%)	
II	10 (43.5%)	
III	3 (13.0%)	
	Mean (SD)	Median (IQR)
Age (years)	47.8 (19.3)	47.0 (32.0)
Preoperative		
Inlet ratio	0.90 (0.07)	0.91 (0.11)
Outlet ratio	0.96 (0.10)	0.85 (0.10)
Deformity index	0.04 (0.04)	0.04 (0.05)
Asymmetry	12.6 (11.0)	10.6 (13.1)
Deformity ratio	0.94 (0.10)	0.94 (0.10)
VD	14.9 (14.7)	12.6 (13.3)
HD	13.0 (13.7)	6.65 (18.3)
Postoperative		
Inlet ratio	0.92 (0.07)	0.92 (0.06)
Outlet ratio	0.91 (0.08)	0.92 (0.11)
Deformity index	0.05 (0.06)	0.04 (0.05)
Asymmetry	11.9 (9.71)	10.1 (15.0)
Deformity ratio	0.91 (0.10)	0.93 (0.10)
VD	6.99 (11.1)	4.33 (9.90)
HD	7.33 (8.75)	3.81 (13.4)
VD change	7.46 (5.22)	7.73 (7.12)
HD change	7.63 (5.25)	7.95 (7.33)

Abbreviations: SD = standard deviation, IQR = interquartile range, VD = vertical displacement, and HD = horizontal displacement.

**Table 3:** Distribution of outcomes among time windows.

	EQ−5D−5L	EQ−VAS	Majeed Pelvic Score
Variable	Mean (SD)	Mean (SD)	Mean (SD)
Time window			
6–8 weeks	0.14 (0.43)	51.2 (11.7)	49.2 (9.32)
3 months	0.46 (0.28)	63.7 (15.9)	60.1 (13.2)
6 months	0.74 (0.16)	77.5 (15.9)	77.5 (15.2)
1 year	0.94 (0.09)	92.3 (9.32)	94.5 (8.12)
Crude estimated (95% CI)	0.32 (0.25, 0.39)	0.17 (0.14, 0.20)	0.20 (0.18, 0.22)
*p*-value	<0.0001	<0.0001	<0.0001
* Adjusted estimated (95% CI)	0.31 (0.25, 0.37)	0.17 (0.14, 0.20)	0.20 (0.18, 0.22)
* *p*-value	<0.0001	<0.0001	<0.0001

* Adjusted for age and gender in the GEE model. Abbreviations: SD = standard deviation, CI = confidence interval, and GEE = Generalized estimating equations.

**Table 4:** Association between EQ−5D−5L scores and measurements in the GEE model.

Variable	Crude Estimated (95% CI)	*p*-Value	* Adjusted Estimated (95% CI)	** *p*-Value
Preoperative				
Inlet ratio	0.948 (−1.492, 3.388)	0.446	1.013 (−0.275, 2.300)	0.123
Outlet ratio	2.409 (0.838, 3.981)	0.003	2.141 (0.041, 4.241)	0.046 **
Deformity index	1.530 (−0.359, 3.419)	0.112	0.614 (−1.428, 2.656)	0.556
Asymmetry	0.005 (−0.005, 0.014)	0.327	0.002 (−0.007, 0.010)	0.728
Deformity ratio	0.344 (−0.936, 1.623)	0.599	−0.314 (−1.121, 0.493)	0.446
VD	0.002 (−0.002, 0.007)	0.371	−0.002 (−0.006, 0.003)	0.461
HD	−0.001 (−0.009, 0.008)	0.845	−0.002 (−0.010, 0.007)	0.673
Postoperative				
Inlet ratio	1.157 (−1.204, 3.519)	0.337	1.359 (0.144, 2.574)	0.028 **
Outlet ratio	1.605 (0.184 3.026)	0.027	1.804 (1.301, 2.307)	<0.0001 **
Deformity index	0.755 (−0.219, 1.728)	0.129	−0.994 (−2.093, 0.106)	0.077
Asymmetry	−0.007 (−0.019, 0.006)	0.321	−0.008 (−0.019, 0.003)	0.144
Deformity ratio	−0.350 (−1.105, 0.405)	0.363	0.651 (−0.005, 1.301)	0.052
VD	0.003 (−0.003, 0.009)	0.365	−0.004 (−0.010, 0.002)	0.215
HD	−0.009 (−0.020, 0.002)	0.105	−0.010 (−0.018, −0.002)	0.010 **
VD change	0.009 (−0.011, 0.029)	0.388	0.008 (−0.008, 0.023)	0.331
HD change	0.006 (−0.014, 0.027)	0.544	0.007 (−0.009, 0.022)	0.402

* Adjusted for age, sex, and time window. ** *p*-values < 0.05 represent statistical significance. Abbreviations: GEE = Generalized estimating equations, CI = confidence interval, VD = vertical displacement, and HD = horizontal displacement.

**Table 5:** Associations between the EQ−VAS score and measurements in the GEE model.

Variable	Crude Estimated (95% CI)	*p*-Value	* Adjusted Estimated (95% CI)	** *p*-Value
Preoperative				
Inlet ratio	0.733 (−1.173, 2.640)	0.451	0.463 (−0.393, 1.318)	0.289.
Outlet ratio	1.299 (0.591, 2.008)	0.0003	0.330 (−0.240, 0.900)	0.256
Deformity index	1.122 (−0.830, 3.073)	0.260	0.623 (−1.076, 2.322)	0.472
Asymmetry	0.002 (−0.009, 0.013)	0.700	0.001 (−0.008, 0.009)	0.822
Deformity ratio	0.464 (−0.881, 1.810)	0.499	−0.308 (−0.957, 0.341)	0.353
VD	0.003 (−0.004, 0.009)	0.453	−0.0007 (−0.004, 0.03)	0.693
HD	−0.002 (−0.008, 0.005)	0.593	−0.002 (−0.007, 0.003)	0.377
Postoperative				
Inlet ratio	1.233 (−0.879, 3.345)	0.252	1.270 (0.093, 2.447)	0.034 **
Outlet ratio	0.551 (−0.047, 1.549)	0.279	0.455 (−0.365, 1.274)	0.277
Deformity index	1.027 (0.017, 2.037)	0.046	−0.383 (−1.042, 0.276)	0.255
Asymmetry	−0.004 (−0.014, 0.006)	0.432	−0.003 (−0.009, 0.004)	0.459
Deformity ratio	−0.460 (−1.316, 0.396)	0.292	0.203 (−0.184, 0.590)	0.303
VD	0.005 (−0.002, 0.011)	0.197	−0.0002 (−0.004, 0.003)	0.927
HD	−0.003 (−0.009, 0.004)	0.436	−0.004 (−0.010, 0.001)	0.130
VD change	−0.003 (−0.026, 0.019)	0.779	−0.005 (−0.022, 0.012)	0.572
HD change	−0.005 (−0.027, 0.018)	0.688	−0.005 (−0.023, 0.012)	0.555

* Adjusted for age, sex, and time window; ** *p*-values < 0.05, representing statistical significance. Abbreviations: GEE = Generalized estimating equations, CI = confidence interval, VD = vertical displacement, and HD = horizontal displacement.

**Table 6:** Association between the Majeed Pelvic Score and measurements in the GEE model.

Variable	Crude Estimated (95% CI)	*p*-Value	* Adjusted Estimated (95% CI)	** *p*-Value
Preoperative				
Inlet ratio	0.937 (−0.463, 2.337)	0.190	0.714 (−0.134, 1.562)	0.099
outlet ratio	0.790 (0.038, 1.542)	0.040	0.009 (−0.579, 0.600)	0.977
Deformity index	0.618 (−1.354, 2.589)	0.539	0.030 (−1.932, 1.991)	0.976
Asymmetry	0.0004 (−0.010, 0.011)	0.942	−0.001 (−0.010, 0.008)	0.763
Deformity ratio	0.168 (−0.772, 1.108)	0.726	−0.207 (−0.811, 0.396)	0.501
VD	0.0004 (−0.004, 0.004)	0.856	−0.001 (−0004, 0.003)	0.586
HD	−0.001 (−0.008, 0.006)	0.868	−0.001 (−0.007, 0.005)	0.716
Postoperative				
Inlet ratio	1.126 (−0.414, 2.665)	0.152	0.871 (−0.376, 2.117)	0.171
outlet ratio	0.528 (−0.238, 1.294)	0.177	0.342 (−0.422, 1.105)	0.381
Deformity index	0.186 (−0.628, 1.000)	0.654	−0.641 (−1.415, 0.133)	0.105
Asymmetry	−0.005 (−0.013, 0.003)	0.201	−0.003 (−0.010, 0.003)	0.322
Deformity ratio	−0.038 (−0.612, 0.535)	0.896	0.365 (−0.090, 0.820)	0.115
VD	0.001 (−0.003, 0.005)	0.582	−0.001 (−0.005, 0.002)	0.441
HD	−0.004 (−0.010, 0.002)	0.171	−0.004 (−0.010, 0.002)	0.199
VD change	−0.002 (−0.025, 0.021)	0876	−0.003 (−0.023, 0.017)	0.794
HD change	−0.003 (−0.026, 0.020)	0.814	−0.003 (−0.023, 0.018)	0.798

* Adjusted for age, sex, and time window; ** *p*-values < 0.05, representing statistical significance. Abbreviations: GEE = Generalized estimating equations, CI = confidence interval, VD = vertical displacement, and HD = horizontal displacement.

## Data Availability

Not applicable.
